# Do Implantable Cardioverter-Defibrillators Prevent Sudden Cardiac Death in End-Stage Renal Disease Patients on Dialysis?

**DOI:** 10.3390/jcm13041176

**Published:** 2024-02-19

**Authors:** Beata Franczyk, Jacek Rysz, Robert Olszewski, Anna Gluba-Sagr

**Affiliations:** 1 Department of Nephrology, Hypertension and Family Medicine, Medical University of Lodz, 90-419 Lodz, Poland; beata.franczyk-skora@umed.lodz.pl (B.F.); jacek.rysz@umed.lodz.pl (J.R.); 2 Department of Gerontology, Public Health and Didactics, National Institute of Geriatrics, Rheumatology and Rehabilitation, 02-637 Warsaw, Poland; robert.olszewski@me.com

**Keywords:** end-stage renal disease, sudden cardiac death, implantable cardioverter-defibrillator

## Abstract

Chronic kidney disease patients appear to be predisposed to heart rhythm disorders, including atrial fibrillation/atrial flutter, ventricular arrhythmias, and supraventricular tachycardias, which increase the risk of sudden cardiac death. The pathophysiological factors underlying arrhythmia and sudden cardiac death in patients with end-stage renal disease are unique and include timing and frequency of dialysis and dialysate composition, vulnerable myocardium, and acute proarrhythmic factors triggering asystole. The high incidence of sudden cardiac deaths suggests that this population could benefit from implantable cardioverter-defibrillator therapy. The introduction of implantable cardioverter-defibrillators significantly decreased the rate of all-cause mortality; however, the benefits of this therapy among patients with chronic kidney disease remain controversial since the studies provide conflicting results. Electrolyte imbalances in haemodialysis patients may result in ineffective shock therapy or the appearance of non-shockable underlying arrhythmic sudden cardiac death. Moreover, the implantation of such devices is associated with a risk of infections and central venous stenosis. Therefore, in the population of patients with heart failure and severe renal impairment, periprocedural risk and life expectancy must be considered when deciding on potential device implantation. Harmonised management of rhythm disorders and renal disease can potentially minimise risks and improve patients’ outcomes and prognosis.

## 1. Introduction

Patients with impaired renal function are more susceptible to heart failure (HF) development due to sodium and fluid retention [1]. Likewise, the presence of HF increases the risk of the development of chronic kidney disease (CKD) and progression to end-stage renal disease (ESRD) as a result of impaired renal haemodynamics [2,3,4]. The presence of advanced CKD accompanied by HF, especially HF with reduced ejection fraction (HFrEF), increases the risk of mortality [5]. Cardiovascular diseases (CADs) are diagnosed in about 50% of dialysis patients, and relative cardiovascular mortality in this population is 20 times higher compared with the general population [6]. Moreover, approximately 25% of all-cause deaths in the population of dialysis patients are due to sudden cardiac death (SCD) [7]. The risk of SCD is four- to twenty-fold higher in CKD patients compared with the general population [8,9,10]. The pathophysiological factors underlying arrhythmia and SCD in patients with ESRD are unique and include, among others, timing and frequency of dialysis and dialysate composition [11]. Fluid overload can also trigger arrhythmias, aggravating the risk of SCD. Dialysis-related risk of SCD could be associated with excessive accumulation followed by aggressive removal of potassium and fluid on the first day of dialysis in the week [12,13]. Moreover, metabolic alkalosis related to exposure to high bicarbonate dialysate may result in hypokalaemia, haemodynamic instability, and QT prolongation [7,14,15]. Therefore, the occurrence of SCD has been reported to be more common on haemodialysis days, particularly on Mondays and Tuesdays after the long dialysis-free period [16,17].

Apart from dialysis-related factors, the occurrence of SCD may be associated with vulnerable myocardium (coronary artery and heart structure pathology) as well as the presence of acute proarrhythmic factors triggering asystole [7]. CKD patients appear to be predisposed to heart rhythm disorders, including atrial fibrillation (AF)/atrial flutter, ventricular arrhythmias (VAs), and supraventricular tachycardias [18]. This group of patients was found to show diminished heart rate response to subcutaneous nerve activity and conduction system diseases. In CKD, rapid worsening of sympathetic tone precedes atrioventricular block, VA, and sudden death [19,20]. Left ventricular hypertrophy (LVH), which is a common finding in patients on dialysis, also enhances the risk of SCD [21]. In general, ventricular fibrillation (VF) or ventricular tachycardia (VT) are the most common causes of patients’ SCDs.

Indeed, a retrospective study including haemodialysis patients with wearable cardioverter-defibrillators demonstrated that 79% of sudden cardiac arrests were associated with VT or VF [22]. However, the results of another study suggested that in ESRD patients, asystole, not tachyarrhythmias, may be the more frequent type of fatal arrhythmia [23]. Another study of haemodialysis patients with cardiac arrest demonstrated the following causes of cardiac arrest: VT and VF (in 31.6% of patients), bradycardia (26.3%), and asystole (15.8%) [24]. Therefore, the identification of the most frequent terminal arrhythmia in haemodialysis patients is highly required since non-ventricular arrhythmias do not respond to defibrillation [25].

In contrast to the general population, reduced ejection fraction is not so frequently observed in haemodialysis patients experiencing SCD; however, they often suffer from diastolic dysfunction associated with LVH [26]. Despite the high risk of mortality, guideline-directed medical therapy in this group of patients is not so frequently utilised [27]. The high incidence of SCD suggests that this population could benefit from implantable cardioverter-defibrillator (ICD) therapy. However, the benefits of this therapy among patients with CKD remain controversial since the results of studies provide conflicting results. In the analysis of data of patients participating in the Multicenter Automatic Defibrillator Implantation Trial–Cardiac Resynchronization Therapy (MADIT–CRT), patients with moderate GFR were demonstrated not only to be less likely to experience VT/VF but also to receive an appropriate shock from the device when compared with the higher GFR group (for VT/VF 95% CI 0.48–0.88, *p* = 0.005) [28]. According to the authors, this finding may be explained by the presence of arrhythmias in CKD patients that are refractory to ICD therapy and thus are more likely to be fatal. At the same time, in the MADIT-CRT analysis, the risk of death without experiencing VT/VF was higher in patients with moderate CKD (95% CI 2.38–5.12, *p* < 0.001). Patients with moderate CKD and implanted with an ICD or CRT-D device were reported to have fewer nonfatal ventricular tachyarrhythmia events. Thus, these results suggest diminished benefit of the ICD even in patients with moderate CKD, as well as a lower rate of appropriate therapies in patients with moderate CKD [28].

The paragraphs below summarise the benefits and risks of ICD implantation in primary or secondary prevention in CKD patients.

## 2. Implantable Cardioverter-Defibrillators (ICDs) in CKD/ESRD in Primary and Secondary Prevention

Implantable cardioverter-defibrillators (ICDs) represent a recognised therapy whose purpose is to prevent sudden cardiac death [29]. Their introduction has significantly decreased the rate of all-cause mortality, including SCD, in populations [30,31,32,33]. ICDs are indicated for cardiac arrest survivors, patients with severe left ventricular dysfunction resulting from ischaemic or non-ischaemic cardiomyopathy, and individuals with both sustained VAs and structural heart disease [34,35]. According to the guidelines of the European Society of Cardiology (ESC) and ACC/AHA/HFSA, ICDs for primary prevention are recommended for patients meeting all the following criteria: ischaemic HF aetiology, NYHA class II–III symptoms, LVEF ≤ 35%, ≥3 months of guideline-directed medical therapy (ESC) or chronic optimal medical therapy (ACC/AHA/HFSA), more than 40 days from a myocardial infarction (MI), and with expected survival > 1 year [36,37] The ACC/AHA/HFSA guidelines also recommend ICDs for patients with non-ischaemic aetiology, while in the ESC guidelines device implantation in such patients is a IIa A recommendation. ICDs for secondary prevention are recommended in individuals with documented VF or haemodynamically not tolerated VT in the absence of reversible causes [35,38]. Moreover, the ESC guidelines ascribe a class IIb recommendation for wearable cardioverter-defibrillators for selected HF patients who are at high risk for sudden death but in whom ICDs are not suitable or ICDs or wearable devices would serve as a bridge to an implanted device [36].

### 2.1. Primary Prevention

Currently available data concerning the advantages of ICDs in primary prevention in patients with LVEF ≤ 35% and advanced CKD are not encouraging, as a result of the high risk of complications, morbidity, and mortality [18,39]. The results of sparse observational studies have confirmed that ICD implantation as a primary prevention measure could have no or even adverse impact on mortality in ESRD patients, especially dialysis-dependent ones [40,41,42]. The comparative study using propensity score techniques to decrease confounding factors failed to show significant survival advantage associated with ICDs [HR 0.87, 95% confidence interval (CI) 0.66–1.13, log-rank *p* = 0.29] in 303 haemodialysis patients with HF who received ICDs for primary prevention compared with matched controls without ICDs [43]. One-year (three-year) mortality was 42.2% (68.8%) in the dialysis ICD recipients compared with 38.1% (75.7%) in the control group [43]. Also, a prospective randomised study of dialysis patients with LVEF ≥35% and without a class I ICD indication demonstrated that ICD implantation did not diminish SCD rate or all-cause mortality [44]. In this study, the observed cumulative incidence of SCD was 9.7% in the group implanted with ICDs versus 7.9% in the control group [hazard ratio (HR) 1.32 (95% CI 0.53–3.29, *p* = 0.55)], and the 5-year mortality was high in both groups (50.6% vs. 54.5%). According to the authors, the failure to decrease patients’ mortality with ICD implantation was associated among others with ineffective termination of shocks or immediate reinitiation after shock delivery stemming either from the occurrence of non-shockable rhythms or arrhythmia triggered in a state of hyperkalaemia and/or severe acid–base balance disorders [17,45]. Another study demonstrated that in-hospital mortality after ICD placement in HD patients was nearly five-fold higher compared with non-dialysis patients [46]. Decreased survival of ESRD patients may be associated with the lack or inappropriate treatment of HF following ICD implantation. According to reports, ESRD patients frequently do not receive or receive inadequate guideline-directed medical therapy with beta-blockers, angiotensin-receptor blockers, and angiotensin-converting-enzyme inhibitors accompanying ICD placement due to the presence of many comorbidities, including diabetes mellitus, ischaemic cardiac disease, and chronic HF [47,48]. Moreover, CKD was suggested to be the strongest predictor of all-cause mortality in patients implanted with ICD for primary prevention of SCD (multivariate adjusted HR of 2.08, *p* = 0.001) [49]. However, Hage et al. [49] suggested that in primary prevention, CKD patients displayed a higher probability of appropriate ICD therapy (multivariate adjusted HR 3.53, *p* < 0.0001); therefore, they implied that individuals with CKD may benefit from ICD implantation to a greater extent than non-CKD patients. The results of recent studies have demonstrated a high rate of asystole-related deaths in HD patients, but it seems that ICDs with backup pacing should also be beneficial in these patients [48,50]. Therefore, it appears that the lack of effectiveness of ICDs in primary prevention in HD patients may be associated with different problems.

However, there are also studies which demonstrated the benefits of ICD implantation for primary prevention in CKD patients. The DEFINITE (Defibrillators in Non-Ischemic Cardiomyopathy Treatment Evaluation) trial evaluated the effects of ICD in primary prevention in patients with non-ischaemic heart disease, LVEF ≤ 35%, and premature ventricular complexes or nonsustained ventricular tachycardia (NSVT). A statistically significant decrease in the SCD rate, but no improvement in the all-cause mortality rate, was observed after a mean follow-up of 29 months [31]. Furthermore, the results of the analysis of data from the multicentre registry demonstrated that in ESRD patients with ventricular dysfunction (LVEF < 35%), the implantation of ICDs for primary prevention was associated with a higher overall survival rate [HR 0.40 (95% CI 0.19–0.82)] compared with individuals without ICDs [51]. The differences in outcomes may be associated with the choice of control group. In the analysis of the multicentre registry, patients with normal renal function were included in the control group.

Moreover, some studies suggested that benefits related to the primary prevention of SCD in CKD patients may depend on the patient’s age and stage of kidney disease. A meta-analysis of 2867 subjects enrolled in primary prevention ICD trials and controls demonstrated that the reduction in mortality following ICD therapy strongly depended on baseline kidney function [8]. The survival benefit of ICDs is much more visible in non-dialysis patients compared with dialysis patients [52]. In the large Multicenter Automatic Defibrillator Implantation Trial (MADIT-II), ICDs were associated with greater survival in patients with mild to moderate or no renal disease; however, in patients with more advanced renal disease there were no significant benefits from this treatment [53]. The implantation of ICDs translated into a survival benefit in each eGFR category ≥ 35 mL/min/1.73 m^2^. The overall risk was reduced by 32% for all-cause mortality (*p* = 0.01) and by 66% for SCD (*p* < 0.001). In contrast, no ICD-related benefit was observed in a group of patients with eGFR < 35 mL/min/1.73 m^2^ (all-cause mortality HR 1.09, *p* = 0.84; SCD HR 0.95, *p* = 0.95) in this trial [53]. In turn, the results of a meta-analysis including three trials demonstrated that ICD implantation was associated with a significant decrease in arrhythmic death only among patients with eGFR ≥ 60 mL/min/1.73 m^2^, but not in those with eGFR < 60 mL/min/1.73 m^2^, who did not significantly benefit from ICD use with primary prevention ICDs [8]. Also, Hess et al. [40] demonstrated that mortality risk following ICD placement as primary prevention was proportional to CKD severity. However, it is not possible to define any “threshold” eGFR at which the mortality benefit of ICD implantation compared with usual care is lost, due to the small number of patients with advanced CKD in the aforementioned trials. Amin et al. [54] observed that the beneficial effects of ICD implantation for primary prevention of SCD in patients with more advanced renal impairment are less visible and become age-dependent. According to the authors, ICD implantation is advantageous at ages < 80 in patients with stage 3, ages < 75 for stage 4, and ages < 65 for stage 5 [54]. In turn, the analysis of patient-level datasets from MADIT I and II, DEFINITE, and SCD-HeFT (the Sudden Cardiac Death in Heart Failure Trial) revealed that ICD implantation translated into a significant improvement in survival of patients with a low number of comorbidities (<2) (unadjusted HR: 0.59; 95% confidence interval [CI]: 0.40 to 0.87) [55]. However, the benefit was less pronounced in patients with a high number of comorbid illnesses (≥2) (unadjusted HR: 0.71; 95% CI: 0.61 to 0.84). Observed comorbid diseases included chronic kidney disease, ischaemic heart disease, atrial fibrillation, diabetes, peripheral vascular disease, and pulmonary disease [55].

Current guidelines do not offer any recommendations concerning the implantation of ICDs as a primary prevention in patients with various eGFR or kidney impairment levels [56]. Therefore, there is an urgent need to identify patients who can with high probability benefit from an ICD [57]. Due to conflicting data on the benefits of such treatment, only a small percentage of ESRD patients are receiving ICDs.

### 2.2. Secondary Prevention

More convincing results favouring ICD implantation in patients with CKD including ESRD/HD come from the studies in which ICDs were used as secondary prevention. Data collected from participants included in the NCDR ICD Registry who received an ICD revealed that a vast majority of them had a documented guideline-defined secondary prevention indication, especially documented SCD or sustained VT (78%) [58]. Mortality of patients implanted with ICDs as secondary prevention amounted approximately to 10% at 1 year [50,59,60]. The retrospective analysis of a large population of dialysis patients (460 patients with ICDs and 5582 patients without ICDs) with VF/cardiac arrest revealed an independent association between ICD implantation and a 42% reduction in death risk [relative risk (RR) 0.58 (95% CI 0.50, 0.66)] [61]. However, the percentage of ICDs implanted in this study was very low (8%). Since only 10% of the entire cohort was subjected to diagnostic electrophysiologic tests, the authors suggested that the reason for not implanting an ICD was not associated with a negative finding on such diagnostic tests. The authors observed that women and the Black population were less likely to receive ICD therapy. Also, Charytan et al. [62] observed lower overall mortality risk in patients implanted with ICDs as secondary prevention (14%, 95% CI, 9–19%) compared with propensity-matched controls; however, this benefit appeared to be limited to the early period post-implantation. According to the authors, this finding could be ascribed to the fact that ICD implantation may delay arrhythmia-related mortality; however, death eventually occurs, which eliminates the putative benefits of ICD implantation in this high-risk population.

The possible benefits of ICD implantation in patients with advanced kidney disease are difficult to assess since the majority of randomised trials usually excluded this group [8]. Dialysis-related factors may increase the probability of heart blocks, bradycardias, and primary pulseless electrical activity that is defibrillation-resistant. Due to the high risk of nonarrhythmic causes in dialysis patients, the implantation of ICDs may not reduce mortality in some patients [62]. The risk of nonarrhythmic death in the SOLVD study was found to be proportional to worsening CKD, and it was especially high in patients with advanced heart failure [63]. A higher prevalence of nonarrhythmic mortality in patients with GFR < 35 compared with the group with GFR > 35 was also observed in the MADIT-II study [53].

Many studies analysed the effects of ICD implantation for both primary and secondary prevention settings. A study based on Danish nationwide registries (2000–2017) which assessed risk factors associated with increased 1-year mortality in 14,516 patients undergoing first-time ICD implantation for primary or secondary prevention demonstrated that dialysis [odds ratio (OR): 3.26, confidence interval (CI): 2.37–4.49], chronic renal disease (OR: 2.14, CI: 1.66–2.76), cancer (OR: 1.51, CI: 1.15–1.99), age of 70–79 years (OR: 1.65, CI: 1.36–2.01), and age ≥ 80 years (OR: 2.84, CI: 2.15–3.77) were the most important ones [57]. In a study of 696 patients who were implanted with ICDs for clinical reasons (59% primary, 41% secondary prevention), the presence of CKD was associated with higher mortality compared with patients with no CKD in both the primary (43% vs. 15%, *p* < 0.001) and the secondary prevention (37% vs. 23%, *p* = 0.003) groups [49]. In this study, the adjustment for age, gender, and multiple risk factors in the analysis revealed that CKD was independently associated with ICD therapy in the primary prevention group (HR 3.53 [1.75–7.10], *p* < 0.0001) but not in the secondary prevention group (HR 0.63 [0.35–1.13], 0.2). Since in secondary prevention the risk of death and appropriate ICD therapy did not differ between patients with and without CKD, it therefore seems that CKD should not be a factor taken into account when deciding about ICD implantation in patients with a history of SCD. Following the adjustment for age and various covariates, the link between CKD and overall mortality disappeared, which implies that patients with a history of VT/VF are at high risk and CKD status does not further increase the risk [49]. Based on data from 11 studies and 20,196 CKD patients, Fu et al. [19] demonstrated that implantation of ICDs for primary (7 studies) and secondary (4 studies) prevention decreased all-cause mortality in stage 3 CKD patients compared with patients without an ICD device (aHR = 0.71; 95% CI, 0.61 to 0.82). Such a survival benefit was not observed in stage 4 or stage 5 CKD patients. According to the authors, the obtained results may be partly explained by the fact that stage 4 CKD patients are less responsive to ICD implantation as a result of a rise in defibrillation thresholds in patients with worsening renal failure. Higher defibrillation thresholds in most patients implanted with ICDs may be the cause of a higher rate of mortality due to arrhythmias [64]. This finding may provide the explanation for a high incidence of arrhythmic deaths in ESRD patients who underwent ICD implantation [65]. Wase et al. [66] observed the following growing defibrillation thresholds in patients with aggravating kidney function: 11.96 ± 4.56 J in patients with 1–2 CKD, 14.51 ± 5.16 J in stages 3–4 CKD, and 16.33 ± 5.3 J in those with stage 5 CKD/ESRD. In turn, Cheema et al. [65] suggested that numerous factors, including age, diabetes mellitus, ICD type, and concomitant guideline-directed medical treatment may negatively or positively affect the effects of ICD implantation (for primary and secondary prevention) in ESRD patients. ICD insertion for primary prevention was more effective in averting fatal events than insertion for secondary prevention in this nonrandomised study. In the opinion of Fu et al. [19], a high rate of post-implantation complications, including device-related infections and non-cardiac-related deaths, may negatively impact the survival benefit associated with ICD implantation [19]. In turn, the data from the National Inpatient Sample (NIS) collected in the years 2016–2019 demonstrated that the risk of mortality was not increased in dialysis patients following the implantation of ICDs [67]. The risk of postprocedural complications, such as bleeding or infections, did not rise either. Therefore, the authors suggested the underutilisation of ICD device implantation as means of secondary prevention in HD patients with end-stage heart failure and accompanying ESRD. However, other studies suggested that the beneficial impact of ICDs on patients could be compromised by competing causes of death, such as infections, nonarrhythmic cardiac death, and cancers. This may outweigh the benefits of ICDs related to the prevention of arrhythmic mortality. Moreover, ICD recipients with CKD were found to have decreased survival compared with their non-CKD counterparts, which translated into shortened ICD exposure time and subsequently into diminished opportunity for ICDs to prevent arrhythmic death [8,47,68,69].

The impact of the type of ICD on patients’ outcomes has also been assessed. Katz et al. [58] demonstrated that ICD type (single-chamber vs. dual-chamber vs. biventricular) was not related to differences in mortality rate at 3 months or at 1 year; however, at 2 years, the implantation of dual-chamber ICDs was found to be associated with a lower risk of mortality (HR: 0.89; 95% CI: 0.84 to 0.94) compared with implantation of a single-chamber device. During 11 years of follow-up, Cheema et al. [65] observed that the implantation of dual-chamber and biventricular ICDs compared with single-chamber ICDs translated into improved survival, which could be associated with the beneficial impact of dual pacing on chronic atrial fibrillation (thus probably increasing survival of such patients compared with those with single-chamber ICDs) as well as with the harmful effect of chronic right ventricle (RV) pacing that is usually avoided or limited with a dual-chamber ICD compared with a single-chamber ICD [70]. Biventricular ICDs were found to be a stronger predictor of improved survival in comparison with dual-chamber ICDs [70]. Cheema et al. [65] demonstrated that the correction of ventricular dyssynchrony in patients with HF via either biventricular pacing or cardiac resynchronisation therapy ameliorated patient survival rates and other clinical outcomes [71]. Moreover, they reported a higher mortality rate in patients with ICDs implanted for secondary prevention compared with those with primary prevention ICDs. This finding may suggest that an earlier procedure performed at the stage of cardiomyopathy in a patient already on the continuum of CKD (primary prevention) is more effective than ICD implantation due to cardiac arrhythmia or arrest. The authors also underlined that apart from the risks related to the ICD implantation itself, the presence of CKD and unique risk factors for this group (especially patients with moderate to severe CKD), including the presence of uraemic toxins, increased inflammation–malnutrition state, anaemia, hyperhomocysteinaemia, higher calcium intake, and abnormalities in bone mineral metabolism, may contribute to greater total and arrhythmic mortality in CKD patients [72]. Moreover, CKD is a risk factor for cardiovascular disease [73]. Furthermore, other traditional cardiovascular risk factors, such as diabetes mellitus, metabolic syndrome, dyslipidaemia, and advanced age, are highly prevailing in this group of patients [74,75]. CKD patients show a tendency towards enhanced sympathetic activity and arrhythmias, and following ICD implantation, higher defibrillation thresholds are reported in this group [76]. All these factors contribute to higher mortality of CKD patients, despite the presence of ICDs. However, Tompkins et al. [77] demonstrated that the exclusion of very sick patients and those with major diseases which required treatment from their randomised study did not change the mortality in patients implanted with an ICD. The observed differences in patients’ mortality in various studies could be related to the choice of various cohorts and comparators, as well as different uses of propensity scores.

## 3. Complications Related to Transvenous ICDs

The high rate of deaths following ICD implantation (as aforementioned) could be partly attributed to infections, followed by SCD. Observational studies have reported a higher rate of complications among CKD (especially ESRD) recipients of ICDs for primary and secondary prevention compared with patients with preserved renal function [47,48,68]. The prospective randomised study of dialysis patients revealed that the frequency of implantable device-related adverse events was as high as 27.5% and included procedure-related events (such as infections and haematomas) and lead dysfunction [44]. The frequency of bacteraemia in dialysis patients following ICD implantation was high (7.5% of cases) and led to the necessity of ICD explantation [44]. Uraemic state, impaired functioning of the immune system, and coagulopathy were suggested to underlie increased vulnerability to device-related complications in CKD/ESRD patients [78].

### 3.1. Infections

The presence of venous HD catheters and ICDs is associated with a higher risk of endovascular infections and symptomatic central venous stenosis [79]. In the case of ICD infection, the removal of the implanted system is necessary, which is associated with additional risk and possible complications [80]. Infections can stem from the contamination of pocket tissues, device generators during implantation, or blood-related seeding of device leads or cardiac valves from a distant infection site [81]. Susceptibility of dialysis patients to ICD infections can be due to frequent bloodstream access and the use of dialysis catheters [82]. In such patients, strategies to shorten the duration of venous catheter access and to decrease risks for infectious complications should be introduced. The increased occurrence of post-implantation infectious complications in ESRD has been demonstrated in a systematic review and meta-analysis of sixty studies [83]. Infections prolong hospital stays and increase in-hospital mortality [84]. The majority of infections from cardiovascular implantable electronic devices (CIEDs) are related to gram-positive bacteria, including coagulase-negative *Staphylococci* species and *Staphylococcus aureus* [81]. The occurrence of infections after implantation was found to be particularly high in the first year and was also greater in those with diabetes and recent infection [62]. El-Chami et al. [85] observed that apart from dialysis and diabetes mellitus, younger age, valvular disease, anaemia, drug abuse, and depression also considerably enhanced the risk of infection in the multivariable model. However, when they analysed this risk in patients who had ICDs implanted for over a year, only age < 70 years, ESRD with dialysis, and anaemia were found to play the most important roles. In general, the odds for device infection were 25% in dialysis patients, but the risk increased to 125% when only late infections were included in the analysis. This finding may imply that apart from infections related to the surgical procedure (a complication of ICD implantation), ESRD patients on dialysis also face the risk of late infections. The authors suggested that repetitive use of arterial–venous fistula access during dialysis sessions may infuse blood-borne pathogens which may travel within the vasculature and grow on distal devices including ICDs. The rate of infection-related mortality of dialysis patients was assessed in a retrospective study. In this analysis, it was found that approximately 11.3% of dialysis patients die from infection after a mean of 1.4 years from device placement [62]. Another large study including over 9500 haemodialysis patients with ICDs demonstrated that bacteraemia occurred in 52% of patients annually, while device infections occurred in 4.2% [62]. ESRD patients also suffer from pocket infections. In an analysis of a cohort of patients implanted with ICDs, the rate of pocket infections in ESRD patients (7.3%) was higher compared with the non-ESRD group; however, the difference was not statistically significant (*p* = 0.1) [86]. The observed trend could be associated with uraemia-related immune dysfunction as well as transient bacteraemia due to frequent venous access during dialysis [87]. Usually, ICD infections require the removal of the whole system and the introduction of intravenous antibiotics [88]. In the case of lead-associated endocarditis, not only the ICD but frequently also vascular access require extraction [89]. The removal of transvenous leads significantly enhances complication rates, morbidity, and mortality [90,91].

The risk of infections can be decreased by implementing preventive measures, such as the use of epicardial leads which are not exposed to the bloodstream, or the choice of subcutaneous or wearable defibrillators [92,93]. Since the subcutaneous ICDs evade vascular exposure, their use appears to decrease the risk of distant, vascular pathogen exposure-related complications. To limit the number of surgical site infections, novel devices, such as antimicrobial pouches, have been introduced. Moreover, preoperative prophylactic intravenous antibiotics were demonstrated to reduce the occurrence of infections; however, no long-term solutions have been approved in this group of patients [94]. The use of prophylactic systemic antibiotics was found to be associated with a 40–95% relative risk reduction [83]. Administered antibiotics should at least target S. aureus species, but not necessarily methicillin-resistant *S. aureus* (MRSA) [95]. The decision on the usage of antibiotics covering the latter species should be made based on the prevalence of this strain in implanting institutions as well as patient risk. Frequently used antibiotics include flucloxacillin (1–2 g) (iv), first-generation cephalosporins such as cefazolin (1–2 g), and vancomycin (15 mg/kg) in case of allergy to cephalosporins [95]. The administration of these drugs must be completed within 1 h of incision to ensure optimal tissue levels. Periprocedural procedures may include an antibacterial mesh envelope that locally releases, e.g., minocycline and rifampin for a minimum of 7 days to protect against infection development. However, in the opinion of the European Heart Rhythm Association (EHRA), “envelopes” (bio-scaffold or pericardium patches), antibiotic-soaked gauze, and similar products have not been thoroughly studied therefore, and their use is not recommended [95]. Also, the standard use of post-implant antibiotics is not recommended since the results of studies failed to confirm the effectiveness of such an approach. Apart from antibiotic administration, other preventive measures for CIED infections should also be implemented. The protocols for infection control require the confirmation of ICD indication and patient’s health (absence of infections), avoidance of temporary transvenous pacing and central venous lines, prevention of pocket haematomas, avoidance of heparin products perioperatively, continued warfarin treatment at the time of ICD surgery, ensuring the sterility of the operating room, preprocedural disinfection of patients’ skin, hair removal, periprocedural use of adhesive iodophor-impregnated incise drapes, etc. [95,96].

### 3.2. Haematoma

Site haematoma is a frequent complication of ICD implantation in ESRD patients that probably results from inappropriate venous access, platelet dysfunction, or coagulopathy related to uraemic state. The risk of such complications can be decreased by the use of absorbable collagen haemostats, gelatine foams, thrombin patches, and pressure dressings [97]. Many patients are on chronic warfarin therapy before the implantation of a pacemaker or ICD to minimise the thromboembolic risk, while those with underlying coronary artery disease receive aspirin, clopidogrel therapy, or both [98]. Intravenous heparin and the combination of aspirin and clopidogrel therapies have been found to raise the risk of pocket haematoma formation in some studies [99,100,101]. Also, in the BRUISE CONTROL study, patients who remained on warfarin had reduced incidence of clinically significant device–pocket haematoma in comparison with those who were administered bridging therapy with heparin (3.5% vs. 16.0%; relative risk (RR), 0.19; 95% Cl, 0.10 to 0.36; *p* < 0.001) [96]. The bridging with heparin is associated with a short period of normal coagulability or hypercoagulability during which the risk of thromboembolism is increased.

### 3.3. Bleeding and Venous Thrombosis

ESRD patients implanted with ICDs also experience bleeding and venous thrombosis [44,77,86]. Venous stenosis and thrombosis may occur in patients in whom the ICD was implanted ipsilaterally to the dialysis catheter [102]. Leads of ICDs may not only cause central vein stenosis but also induce tricuspid regurgitation [103]. A retrospective analysis of 495 patients with transvenous pacemakers revealed that central venous stenosis occurred in 70% of HD patients with an ipsilateral transvenous pacemaker [102]. Compared with non-dialysis patients, the majority of ESRD individuals are symptomatic, which is related to the intense flow in the arteriovenous access [104]. Haemodialysis was shown to aggravate the stenosis. Percutaneous balloon angioplasty and stent placement are standard therapeutic options; however, the extraction of lead is recommended before stent implantation [105]. Moreover, percutaneous balloon angioplasty has a low primary patency [89]. As a preventive measure, in dialysis patients, an arteriovenous fistula formation on the contralateral upper limb for cardiac ICD placement or evading the central vein catheter by using subcutaneous ICDs, epicardial and leadless pacemakers are recommended to limit the rate of complications [103].

### 3.4. Other Complications

ICD recipients may also face lead dislodgement or dysfunction requiring lead adjustment or removal [44,77,86]. Lead dislodgement is another complication observed in ESRD, which involves a change in lead tip position accompanied by the modification of electrical lead parameters. One small study demonstrated the occurrence of such a complication solely in ESRD patients, but not in the control group [86]. Pocket reopening and lead repositioning is the solution in case of early dislodgment, while in late displacements, it is recommended to extract the lead and reimplement it in the appropriate chamber [86].

Also, venous hypertension has been reported in patients with arteriovenous haemodialysis access and ipsilateral ICD leads as a result of a high rate of venous blood return [92,106,107]. It has been suggested that the ligation of the arteriovenous access and flow reduction appear to effectively control this complication.

The complications of ICD implantation as a secondary prevention may include death during in-hospital stay after the procedure [29]. Risk factors in patients with high mortality included advanced age (1.9% vs. 0.5% in patients < 80 years old, *p* < 0.001), female gender (0.95% vs. 0.54% in men, *p* = 0.004), higher NYHA class (0.3% for NYHA II, 0.7% for NYHA III, 3.4% for NYHA IV, *p* < 0.001 for all comparisons), and the need for dialysis [29]. Many patients’ deaths are associated with infections; this was described in detail earlier in this text.

Nephrologists and cardiologists should be aware of risks and thus search for more safe alternatives. Leadless pacemakers may prove beneficial in patients with rapidly progressive chronic kidney disease and high odds for renal failure and patients with glomerular filtration rate < 20 mL/min/1.73 m^2^ to decrease the risk of future complications in case of dialysis [103]. However, such devices should be used in addition to S-ICDs in these patients.

## 4. Identification of Patients Benefiting from ICDs

Due to the fact that the effectiveness of ICD implantation in some groups of patients appears unclear, algorithms should be used for the identification of individuals in whom the benefits will outweigh the risks associated with ICD implantation. Buxton et al. [108] have suggested that the induction of sustained ventricular tachyarrhythmias during programmed ventricular stimulation (PVS) may enable the identification of patients at risk of post-MI SCD who may benefit from ICD placement in the population of individuals with nonsustained VT and LVEF between 35% and 40%. The two-step stratification algorithm proposed in the PRESERVE EF study was found to be characterised by 100% sensitivity, 93.8% specificity, 22% positive predictive value, and 100% negative predictive value [109]. The first step involved ambulatory 24 h and signal-averaged electrocardiogram recordings assessed for the presence of specific non-invasive risk factors, including >30 premature ventricular complexes/hour (24 h electrocardiography; 24 h-ECG), the presence of non-sustained VT (24 h-ECG), QTc derived from 24 h-ECG > 440 ms (men) or >450 ms (women), 2/3 positive criteria for late potentials from a signal recorded in three pseudo-orthogonal leads, ambulatory T-wave alternans ≥65 μV in two Holter channels, standard deviation of normal RR intervals ≤ 75 ms (24 h-ECG), deceleration capacity ≤ 4.5 ms, heart rate turbulence onset ≥ 0%, and heart rate turbulence slope ≤ 2.5 ms. In patients with at least one specific non-invasive risk factor, invasive programmed ventricular stimulation (PVS) was performed. Then, patients were stratified into three groups: Group 1 comprised patients without any non-invasive risk factors, Group 2 comprised patients with at least one risk factor who are non-inducible upon PVS, and Group 3 comprised patients with at least one risk factor who are inducible upon PVS. ICD implantation was performed only in the third group. Another study suggested that left ventricle global longitudinal strain (LV GLS) could improve the identification of predialysis and dialysis patients at risk for VAs and SCD [110]. Moreover, the AUC area was larger than that for LVEF, which implies that LV GLS may be a superior marker of arrhythmogenic risk due to the fact that it often reveals LV myocardial damage that LVEF may not be able to detect. Furthermore, the presence of scars or fibrous tissue within viable myocardium was found to increase the nonuniform anisotropy degree and enhance the risk of electric uncoupling and also to be associated with the occurrence of areas of conduction block and slow conduction [111]. These phenomena can be detected with two-dimensional speckle-tracking and the measurement of LV mechanical dispersion. Hensen et al. [110] demonstrated that LV mechanical dispersion was significantly longer in patients with VA or SCD compared with patients without.

## 5. Subcutaneous ICDs

The use of traditional transvenous ICDs has been found to be associated with a high risk of infection and vascular injury (as described above). Therefore, subcutaneous ICDs that are fully extrathoracic and require neither intravascular defibrillator leads nor vascular access have been developed to overcome such problems [34]. This type of ICD was approved by the Food and Drug Administration in 2012 for patients who require ICDs for primary and secondary prevention of sudden cardiac death [90]. However, this device is not intended for patients with indications for permanent pacing, treatment of ventricular tachycardia (antitachycardiac pacing), or bradycardia and pre-existing unipolar pacemaker leads [34,89,112]. The American College of Cardiology (ACC)/American Heart Association (AHA) Task Force on Clinical Practice Guidelines (2017) and the Heart Rhythm Society (HRS) guidelines for the management of patients with ventricular arrhythmias and the prevention of sudden cardiac death [35] suggest the implantation of subcutaneous ICDs (S-ICDs) in patients at high risk of SCD and high risk of infection; however, the definition of patients at high risk of infection has not been provided there.

Patients undergoing HD have problems with vascular access and face a high risk of bacteraemia; therefore, it appears that S-ICDs which circumvent vascular access may be more advantageous for these patients [113]. Due to the fact that S-ICDs do not transverse central veins, the risk of central vein stenosis is also diminished [48]. Both catheter and cardiac leads of subcutaneous ICDs are placed in separate compartments, not in the bloodstream [62]. Moreover, the implantation of S-ICDs is not complicated and does not require intravascular manipulation or fluoroscopy, which translates into lower risk of complications, including cardiac tamponade, pneumothorax, and haemothorax [34].

In 2018, 20% of all ICD implants in the US were subcutaneous, while their use in patients on dialysis amounted to 70%, even though large-scale studies assessing S-ICDs have not been performed and no definitive data supporting the safety of these devices in patients on dialysis were available [34]. Currently, S-ICDs are used in patients in whom the standard solutions failed, e.g., in those with prior device infection, in patients with complicated venous access, and in individuals who can outlive the transvenous ICD leads [112]. El-Chami et al. [113] compared the effectiveness of subcutaneous ICDs in patients (*n* = 220) on HD at the time of implantation with a group of non-HD individuals (*n* = 1437). At the time of S-ICD implantation, patients in the HD group had a lower ejection fraction compared with the non-HD group (28.6% ± 11.3% vs. 32.6% ± 14.9%; *p* < 0.0001), they had more comorbidities, and they displayed higher mortality (17.4% vs. 3.7%). However, the occurrence of post-implantation complications was similar in the two groups (7.9% and 7.7% during the first year), which suggests the safety of this procedure. Also, in another study including 79 patients with S-ICDs, these devices did not increase the risk of complications in dialysis and non-dialysis patients [114]. In a population of HD patients, the use of subcutaneous ICDs was also not related to excessive inappropriate shocks in comparison with non-dialysis patients [115]. However, some studies have reported that S-ICDs may be associated with a higher rate of in-hospital (perioperative) cardiac arrest [116]. At the time of implantation, S-ICDs require defibrillation threshold testing since these devices need higher energy to effectively convert ventricular fibrillation to normal sinus rhythm [117]. In order for such tests to be performed, patients receive deeper sedation, which could predispose them to haemodynamic compromise and cardiac arrest even if the VF conversion is successful. Indeed, Pun et al. [34] demonstrated that in-hospital cardiac arrest was more frequent in patients with an S-ICD when compared with those with a transvenous ICD (OR 4.72; 95% Cl 1.71 to 11.17, *p* = 0.002). Moreover, 58% of patients with S-ICDs who had in-hospital cardiac arrest underwent defibrillation threshold testing compared with 20% of patients with transvenous ICDs. Whether S-ICD implantation increases the risks of periprocedural cardiac arrest in patients on dialysis due to defibrillation threshold testing requires closer monitoring and further research. An analysis of the FDA’s Manufacturer and User Facility Device Experience (MAUDE) database concerning S-ICDs revealed that out of 23 nonfatal cardiac arrest events, 10 were associated with defibrillation threshold testing at the time of implantation [118].

The most frequent complications reported in the study of S-ICDs included infections (resulting in system removal) and inappropriate shocks which were primarily attributed to oversensing. Transient ECG waveforms predisposed to T-wave oversensing and inappropriate shocks may be triggered by electrolyte and volume fluctuations [119]. Inappropriate shocks can be managed by system reprogramming, removal, or revision. In order to decrease the risk of T-wave oversensing and inappropriate shocks, HD patients should be monitored both shortly before dialysis and after dialysis [119]. Such control enables the assessment of the impact of ECG tracing on fraction (≥35%) shifts in electrolytes, volume status, and autonomic tone. Another study demonstrated that the incidence of inappropriate shocks in HD and non-HD patients with S-ICDs was similar and amounted to 7–13%, which is in agreement with the available data in the literature [90,120,121]. In this study, S-ICDs cardioverted all VTs and VFs in HD patients. In turn, Pun et al. [34] did not report any intravascular complications (including cardiac perforation, haemothorax, and cardiac tamponade) associated with S-ICD use in a study of over 1500 patients. Lower occurrence of complications while using S-ICDs may translate into a decreased risk of distant complications and counterbalance the costs of S-ICD implantation [90,113].

## 6. Conclusions

SCD poses a serious problem in HD patients [7]. Further large studies are necessary to clarify the pathophysiology of this disease, to determine risk factors, and to enable the development of more effective prevention strategies. The implantation of ICDs is one of the measures to decrease dialysis-related arrhythmic risk. Based on available knowledge, defibrillator therapy appears to be associated with a significant survival benefit among patients with mild to moderate or no renal disease; however, no or little benefit was shown in the population of patients with more advanced renal dysfunction. Patients with CKD should therefore be treated according to the same recommendations that apply to the general population; however, when renal function is significantly reduced, there are no clear data to indicate whether these patients will certainly benefit from ICD therapy. The benefits of such therapy in CKD patients depend on various factors, including eGFR, age, diabetes mellitus, other comorbidities, ICD type, and concomitant guideline-directed medical treatment. Therefore, in the population of patients with HF and severe renal impairment, periprocedural risk and life expectancy must be taken into consideration when deciding on potential ICD implantation [2]. The decision concerning the implantation of ICDs in ESRD patients should be made on the basis of individual evaluation of the risk–benefit ratio performed by both a nephrologist and cardiologist [80]. High mortality of ESRD patients despite implantation of ICDs could be related to ineffective shock therapy or the appearance of non-shockable rhythms (asystole/pulseless electrical activity) underlying arrhythmic SCD [23]. Harmonised management of rhythm disorders and renal disease can potentially minimise risks and improve patients’ outcomes and prognosis [79]. Also, the addition of subcutaneous defibrillators has been suggested to reduce the risk of vascular access complications and infections among haemodialysis patients [7].

### Take-Home Message

- CKD patients appear to be predisposed to heart rhythm disorders, including AF/atrial flutter, VAs, and supraventricular tachycardias;
- The risk of SCD is four- to twenty-fold higher in CKD patients compared with the general population;
- ICDs for primary prevention (according to ESC and ACC/AHA/HFSA) are recommended for patients meeting all the following criteria:
  ✓ Ischemic HF etiology;
  ✓ NYHA class II–III symptoms;
  ✓ LVEF ≤ 35%;
  ✓ ≥3 months of guideline-directed medical therapy (ESC) or chronic optimal medical therapy (ACC/AHA/HFSA);
  ✓ More than 40 days from an MI, and with expected survival > 1 year.
- ICD for secondary prevention is recommended in individuals with documented VF or haemodynamically not tolerated VT in the absence of reversible causes (ESC);
- Current guidelines do not offer any recommendations concerning the implantation of ICDs as primary prevention in patients with various eGFRs or kidney impairments due to conflicting results of studies;
- Defibrillator therapy is associated with a survival benefit among patients with mild to moderate or no renal disease; however, no or little benefit was shown in a population of patients with more advanced renal dysfunction;
- Benefits of such therapy in CKD patients were found to depend on various factors, including eGFR, age, diabetes mellitus, other comorbidities, ICD type, and concomitant guideline-directed medical treatment;
- Beneficial impact of ICD on patients could be compromised by competing causes of death, such as infections, nonarrhythmic cardiac death, and cancers;
- The high rate of deaths following ICD implantation could be partly attributed to infections (especially prevalent in HD patients), followed by SCD, site hematoma, lead dislodgement or dysfunction requiring lead adjustment or its removal, bleedings, and venous thrombosis;
- Implementation of pre- and postoperative measures to control infections contributes to a decrease in infection-related mortality in patients who underwent ICD implantation.

## Data Availability

Not applicable.
